# Common Errors in Diagnosis: The Chest
*A Lecture delivered at the Polyclinic.


**Published:** 1914-06-20

**Authors:** J. E. Squire

**Affiliations:** Consulting Physician to Mount Vernon Hospital


### COMMON ERRORS IN DIAGNOSIS.

### The Chest.*

By J. E. SQUIRE, M.D., C.B.; Consulting Physician to Mount Vernon Hospital.

It is strange there should be an idea that the stethoscope is an instrument requiring but little experience to make one expert in its use. We used it in our student days, and found it so easy to get the "hang" of it, as well as to learn the elementary facts which we can make out by its aid, that we do not associate expertness with it. The difficulty about examination of the chest is that we have to diagnose by hearing, rather than by sight, which latter we are accustomed to trust more than hearing. The sounds do not indicate any disease, but only the physical condition: we must bring to bear our knowledge of morbid states in order to arrive at an interpretation of the physical state. Therefore, it is probable that there is no branch of medicine in which expert assistance is more often needed than in the examination of the chest. During the last year I have examined three thousand patients who had already been examined by other doctors, whose reports were before me. From these I am able to say that much is overlooked and a good deal is inaccurately described.

### Errors Excusable and Otherwise.

Some of the errors are inexcusable, but we cannot expect complete immunity from mistakes. The first cause is incomplete examination, and the second is a mistaken inference from the signs which have been noted. The inexcusable errors are chiefly in the first of these categories. The most serious I have met with is that of giving an opinion as to the nature of a complaint without having examined the chest at all, which is not quite so uncommon as might be imagined. Some examine through the clothing, but you will realise that the slight modifications of respiratory movements, or in the intensity and character of the breath-sounds which occur in early tuberculosis of the lung cannot be detected through the clothing.

Sometimes the practitioner, as soon as he has found sufficient to account for the symptoms, rests content, without troubling about the extent of the disease or its possible complications. The condition of both lungs should be investigated: to diagnose a case as tuberculosis is not of very great value when it comes to treatment and prognosis. There may be found a cavity at one apex, but that may be the result of an old illness. The present symptoms, however, may be due to infection of the opposite lung. Pleural effusions, heart disease, and transposition of viscera have passed unnoticed. In one case laryngeal tuberculosis was held to explain the illness, when an examination of the lungs would have shown that one lung was extensively diseased. Heart disease seems to be one of the commonest complications of lung disease which is not detected. An old-standing lesion of one valve may, by back pressure, cause conditions in the lung which are not normal, so that such a patient may be suffering from shortness of breath, may have cough, and may have brought up some blood; and this, with the patient's unfitness for work, may cause a suspicion of consumption. And if, in addition, you hear crepitant râles, this seems to confirm the diagnosis that it is tuberculosis. But fuller examination shows that the base of one or both lungs was more affected than the apex, and that the heart showed marked signs not only of a valvular lesion, but also want of compensation. In some cases there is heart disease without tuberculosis.

A diseased state sometimes passes unrecognised owing to lack of method in the examination. Do not apply your stethoscope straight away, but first listen to the quiet breathing; then tell the patient to take a deep inspiration. And do not neglect inspection. Follow that by percussion and then auscultation. Compare the inspiratory breath sound with the expiratory, and afterwards specially note the adventitious sounds. Disregard these latter until you have listened to the character of the breathing. I ask the patient to cough and then take a deep breath. There are signs brought out on deep breathing which are not manifest on quiet breathing. Next examine for voice sounds, and if necessary palpate for fremitus. Do not forget to carry out inspection before other methods of examination. The trained eye will detect flattening due to an old cavity, modifications of the respiratory movement in some part of the chest, the absence of movement caused by pleural effusion or pneumothorax, and the position of cardiac or vesicular pulsations.

### Misinterpretation of Signs.

To avoid errors arising from misinterpretation of signs is not so simple. The text-book sign of a disease never fits in with any particular case. The books will give as a sign of fluid in the sac, dulness on percussion, loss of vocal resonance, and fremitus; but with the binaural stethoscope you find the breath sounds can be heard even when there is considerable fluid. Vocal resonance and fremitus can be heard when there is much fluid.

Displacement of organs, such as the heart, is mentioned as a sign of pleural effusion, but with even large effusions there may be no heart displacement. On the other hand, the heart may be displaced by something which is not pleural effusion. Failure to detect even large effusions is one of the common errors in chest examinations. Attempts to determine the nature and cause of effusion lead to mistakes which are pardonable. The only way to determine whether the fluid is serous or purulent

* A Lecture delivered at the Polyclinic.

is to withdraw some and examine it. It is not infrequent for old tuberculosis of the lung to be mistaken for recent and active disease, especially if physical signs are relied upon without giving due weight to symptoms as well. An old latent lesion can be used for malingering by a man who wants a rest or who wants to receive Insurance benefit. The practitioner finds signs of a cavity, and, though the symptoms are not in proportion, he says he will give the patient the benefit of the doubt, and sends him to hospital or sanatorium. Persons may expectorate sputum containing tubercle bacilli, and yet there may not be active tuberculosis present. The dilated tubes in chronic bronchitis may serve as culture tubes for bacteria which arrive in the inspired air.

Sanatoria and Bronchitics.

Bronchitic râles may mask signs of tuberculosis: bubbling râles may be mistaken for the softening associated with tuberculosis. There are always great difficulties regarding chronic bronchitics who afterwards become infected with tubercle. Where there are signs of bronchitis limited to one lung, or to one portion of a lung, it is probably a case of tuberculous bronchitis. I have refused to send bronchitics to sanatoria under the Insurance scheme.

A more subtle cause of error in diagnosis is to be found in the absence of a standard by which we can gauge the sounds heard. The sounds and conditions of the normal chest are not sufficiently presented to students, and there are not many opportunities later, except, perhaps, as physicians to an insurance office. You may usually take it that the abnormal side is most likely to give you prolongation and increased intensity of expiratory breath sounds. Sometimes physiological differences in the two lungs may be such as to lead to the opinion that one is diseased. In a series of observations on healthy chests: in 50 per cent. the percussion note was flatter at the right apex than at the left; the breath sounds were louder on the right in 25 per cent.; the vocal resonance and fremitus more marked in 75 per cent. of the chests on the right side. If the left apex gives a flatter note on percussion than the right this cannot be safely ignored. Increased fremitus and resonance on the left is also significant. This reversion may be due to partial consolidation of the left apex, or to modification of the signs over the right lung. Even in marked lung disease mistakes of this kind are not uncommon. Puerile or exaggerated breathing in a healthy lung compensatory to damaged lung on the other side is sometimes mistaken for consolidation or a cavity; but here a comparison of the relative length of expiratory and inspiratory sounds is important. In compensatory breathing, expiration is shorter in duration and less intense than inspiration. Pectoriloquy is not obtained with some compensatory breathing.

Bronchiectasis may be confused with disease of lung. There is a cavity, and the contents are offensive, so mistakes are not surprising. Incompletely resolved pneumonic consolidation is sometimes mistaken for tuberculosis, and hæmatemesis from gastric ulcer may be regarded as hæmoptysis, and tuberculosis may be considered as the cause of the patient's pallor. Tuberculosis is not the only disease in which hæmoptysis occurs. Stained sputum is not uncommon in chronic bronchitis, and a large hæmorrhage from the lungs is common in heart troubles, and, of course, in malignant disease of lung.

Mistakes in the diagnosis of heart lesions are far from common, and are generally due to relying too much on murmurs as diagnostic signs. Functional murmurs may simulate those due to valvular lesions. Heart and vascular sounds conducted by consolidated lung tissue may suggest a vascular lesion. With regard to cardio-vascular murmurs, where the heart is beating against the chest-wall there may be a thin layer of lung between it and the chest-wall, and in a lax condition of the tissues each beat of the heart may drive air out of that layer sufficiently forcibly to give a whiffing or blowing murmur; and as this occurs at each heart beat a systolic murmur is heard over the apex. This may disappear when the patient holds his breath, but it does not always. This whiffing sound varies in intensity at different stages of the respiratory cycle, not the cardiac. It is always systolic, but at inspiration it may be very loud, while at expiration it may be soft, or may disappear altogether.

